# Supplementary material for: Silicon Reduce Structural Carbon Components and Its Potential to Regulate the Physiological Traits of Plants
Source: Plants (Basel). 2025 Jun 11;14(12):1779. doi: 10.3390/plants14121779 (PMC12196780; doi:10.3390/plants14121779)
Supplement: Supplementary file 1 [file plants-14-01779-s001.zip › plants-3608579-supplementary.pdf]

Table. S1. Average annual precipitation and temperature from 2012–2020 at the experimental site.

Year	Precipitation(mm)	Daily average temperature(°C)	Daily average maximum temperatures(°C)	Daily average minimum temperatures(°C)	Temperature difference(°C)
2012	718.6	2.49	9.28	-2.55	11.83
2013	532.5	2.91	10.29	-2.90	13.19
2014	610.8	2.86	9.57	-2.38	11.95
2015	507.2	2.91	9.84	-3.06	12.90
2016	684.2	2.96	10.03	-2.62	12.65
2017	680.0	3.37	10.12	-2.13	12.25
2018	794.3	3.27	9.77	-1.96	11.73
2019	625.0	3.02	9.82	-2.53	12.35
2020	820.9	2.96	9.47	-2.29	11.76

Table. S2. Soil nutrient staus before Si and P addition

Soil layer (cm)	PH	Oranic matter ($g.kg^{-1}$)	Soil Silicon ($g.kg^{-1}$)	Total nitrogen ($g.kg^{-1}$)	NH_4^+-N ($mg.kg^{-1}$)	NO_3^--N ($mg.kg^{-1}$)	Total phosphorus ($g.kg^{-1}$)	Available phosphorous ($mg.kg^{-1}$)
0-15	6.33	70.52	3.64	3.72	10.67	15.25	0.98	25.27

Table. S3. Changes in the contents of soil nutrient with P and Si addition. Different lower-case letters in the same row mean significant difference at 0.05 level. Values are means of six replicates ± SD. Lowercase letters indicate the significance among different treatments ($P < 0.05$).

Treatment	Soil PH	Soil Oranic matter ($g.kg^{-1}$)	Soil Silicon ($g.kg^{-1}$)	Soil total nitrogen ($g.kg^{-1}$)	Soil NH_4^+-N ($mg.kg^{-1}$)	Soil NO_3^--N ($mg.kg^{-1}$)	Soil total phosphorus ($g.kg^{-1}$)	Soil available phosphorous ($mg.kg^{-1}$)
CK	6.33±0.03a	69.45±1.39a	3.64±0.09b	3.71±0.37a	8.64±0.38a	13.23±0.13b	0.93±0.03f	19.20±1.21h
P1	6.37±0.05a	72.54±1.61a	3.22±0.12c	3.68±0.27a	8.68±0.54a	13.02±0.77b	1.10±0.02d	50.99±1.23f
P2	6.41±0.04a	71.68±2.03a	2.81±0.12d	3.70±0.26a	8.64±0.59a	12.83±0.89b	1.22±0.01c	99.50±1.26d
P3	6.44±0.02a	72.12±2.03a	2.09±0.12e	3.70±0.32a	8.53±0.39a	12.20±1.09b	1.38±0.01b	124.20±1.24b
Si	6.38±0.02a	70.48±3.12a	5.14±0.10a	3.72±0.05a	8.64±0.74a	13.87±1.09a	1.00±0.02ef	20.23±1.20g
P1Si	6.34±0.04a	70.67±3.19a	3.64±0.10b	3.68±0.25a	8.58±0.88a	13.92±1.19a	1.24±0.07c	58.03±4.35e
P2Si	6.39±0.03a	73.84±2.85a	3.30±0.09c	3.72±0.14a	8.65±0.91a	14.11±1.19a	1.33±0.07b	117.80±7.24c
P3Si	6.42±0.02a	71.63±2.68a	2.73±0.09d	3.69±0.15a	8.72±0.43a	13.76±1.21a	1.47±0.01a	156.00±6.29a

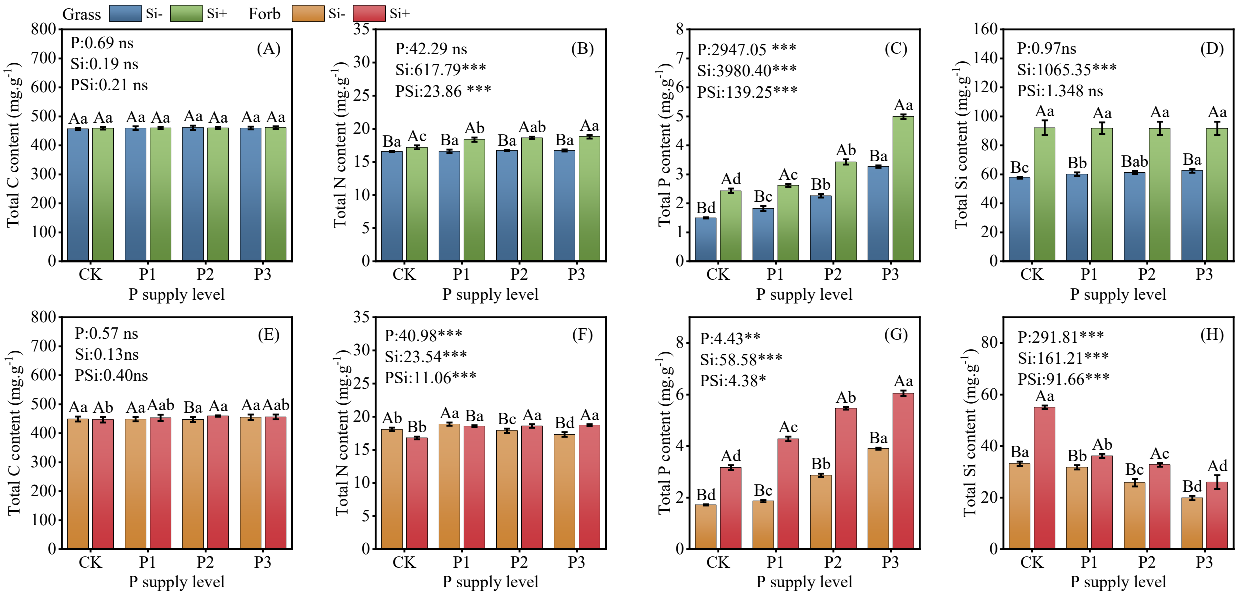

Figure.S1. Effects of phosphorus and silicon addition on the C, N, P and Si concentration in grass and forb leaves. Values are means of six replicates ± SD. The data were analyzed by two-way ANOVA conducted with P, and Si as sources of variation. The significance of the sources of interaction (PxSi) was determined through the P-values: ns, not significant; *$P < 0.05$; **$P < 0.01$; ***$P < 0.001$. Lowercase letters indicate significance between different phosphorus treatment levels ($P < 0.05$), while uppercase letters indicate significance between silicon-added and silicon-removed treatments at the same phosphorus level ($P < 0.05$).
